# Rational Design and Biotechnological Production of Novel AfpB-PAF26 Chimeric Antifungal Proteins

**DOI:** 10.3390/microorganisms6040106

**Published:** 2018-10-15

**Authors:** Marcos Heredero, Sandra Garrigues, Mónica Gandía, Jose F. Marcos, Paloma Manzanares

**Affiliations:** Department of Biotechnology, Instituto de Agroquímica y Tecnología de Alimentos (IATA), Consejo Superior de Investigaciones Científicas (CSIC), Paterna, 46980 Valencia, Spain; marcosherederoiborra@gmail.com (M.H.); sgarrigues@iata.csic.es (S.G.); mgandia@iata.csic.es (M.G.); pmanz@iata.csic.es (P.M.)

**Keywords:** antifungal protein, rational design, chimeric protein, cysteine-rich proteins, *Penicillium digitatum*, FungalBraid

## Abstract

Antimicrobial peptides (AMPs) have been proposed as candidates to develop new antimicrobial compounds for medicine, agriculture, and food preservation. PAF26 is a synthetic antifungal hexapeptide obtained from combinatorial approaches with potent fungicidal activity against filamentous fungi. Other interesting AMPs are the antifungal proteins (AFPs) of fungal origin, which are basic cysteine-rich and small proteins that can be biotechnologically produced in high amounts. A promising AFP is the AfpB identified in the phytopathogen *Penicillium digitatum*. In this work, we aimed to rationally design, biotechnologically produce and test AfpB::PAF26 chimeric proteins to obtain designed AFPs (dAfpBs) with improved properties. The dAfpB6 and dAfpB9 chimeras could be produced using *P. digitatum* as biofactory and a previously described *Penicillium chrysogenum*-based expression cassette, but only dAfpB9 could be purified and characterized. Protein dAfpB9 showed subtle and fungus-dependent differences of fungistatic activity against filamentous fungi compared to native AfpB. Significantly, dAfpB9 lost the fungicidal activity of PAF26 and AfpB, thus disconnecting this activity from the fungistatic activity and mapping fungicidal determinants to the exposed loop L3 of AfpB, wherein modifications are located. This study provides information on the design and development of novel chimeric AFPs.

## 1. Introduction

Fungal infections represent an important risk to human health, food production and safety [1]. In medicine, fungal diseases have increased due to the growing number of immunosuppressive therapies and the appearance of new strains resistant to commonly used antifungal drugs [1,2]. In agriculture, fungi are the main pathogens of crops used for food and feed production. Moreover, mycotoxins produced by some fungi represent a threat for food safety, as they can contaminate food and be dangerous to human health.

Antimicrobial peptides (AMPs) are a broad class of peptides and small proteins that are produced by organisms all along the phylogenetic scale [3,4,5]. AMPs have been proposed as promising candidates for the development of novel antimicrobial compounds. However, despite the great potential of AMPs for multiple applications, some of them present undesired properties that may compromise their real application, such as unspecific toxicity, high susceptibility to degradation, low temperature or pH stability, and low production yields when obtained from their natural sources. One of the attractive attributes of AMPs is their peptidic nature, which would enable their sequence modification by rational design and high-yield production through biotechnology [6,7]. Novel synthetic antifungal peptides with improved properties have been produced through rational design and combinatorial approaches, including fusions of antimicrobial fragments into peptide hybrids, sequence point modifications, de novo rational design based on known AMPs, the identification of minimal motifs that retain full activity, or the incorporation of non-natural amino acids or derivatizations.

Combinatorial peptide chemistry allowed the identification of the antifungal hexapeptide PAF26 (RKKWFW) [8]. PAF26 and PAF26-containing peptides have strong antifungal activity and specifically target and inhibit filamentous fungi including postharvest, plant, and human pathogens in vitro and in vivo [9,10,11,12]. PAF26 is internalized into target fungal cells at sub-inhibitory concentrations in a non-lytic mechanism [13], and has been proposed as a model to investigate the mode of action of cell-penetrating antifungal peptides [14]. Moreover, PAF26 has two functional domains, the N-terminal cationic (RKK) and the C-terminal aromatic (WFW) that allow to differentiate the three steps of its mode of action: (i) interaction with fungal cells; (ii) internalization; and (iii) intracellular killing [15]. Despite these promising properties, the synthetic production of PAF26 for its future application is not affordable, and previous attempts to produce PAF26 biotechnologically have not been successful.

Other AMPs that are of particular interest for their biotechnological applications are the antifungal proteins (AFPs) of fungal origin [16,17]. AFPs can be efficiently produced by biotechnology, which represents a great advantage compared to synthetic peptides in terms of cost and production yields. AFPs are small basic proteins containing multiple cysteine residues that form disulfide bonds and fold into compact structures, conferring a high degree of stability [18,19]. AFPs are translated with a signal peptide (SP) for secretion and a pro-sequence that has been predicted to inactivate the protein until cleavage. Proteins AFP from *Aspergillus giganteus* and PAF from *Penicillium chrysogenum* are the first identified and most studied AFPs to date, and both are naturally secreted by their producer fungus in high yields [20,21]. Recent phylogenetic studies proposed a new classification of AFPs in at least three classes (A, B and C) [22], and a fourth potential class of AFPs has also been identified with mainly anti-yeast activity [23]. In their primary sequence, AFPs contain an amino acid conserved motif, Gly-X-Cys-X_3-9_-Cys, called the γ-core motif [24].

A promising AFP recently described is the class B AFP from *P. digitatum* (AfpB). AfpB was absent in the culture supernatants of *P. digitatum* despite the high gene expression of the corresponding gene [22]. The production of AfpB by *P. digitatum* was finally achieved using a *P. chrysogenum*-based expression cassette, which uses the promoter and terminator of the *paf* gene that naturally directs the production of the *P. chrysogenum* PAF [19,25]. Moreover, the DNA parts of this cassette have been recently adapted to a Golden Gate-based modular cloning platform that allows the straightforward direct cloning of AFP variants [26]. The three-dimensional (3D) structure of AfpB was predicted [27], and shows a similar structural pattern to other experimentally characterized AFPs like PAF [18,28], or PAFB [29] from *P. chrysogenum*. It consists of five antiparallel β-strands connected with three small loops and a big cationic surface-exposed loop. AfpB showed great antifungal efficiency in vitro against a wide range of fungi, including the parental fungus *P. digitatum*, and effectively protected against fungal infection in vivo [30,31]. In addition, AfpB showed a great resistance to high temperatures and proteolysis and showed no cytotoxic effect against human erythrocytes [19].

Given the strong antifungal and fungicidal activity of PAF26, the potential of AfpB as an antifungal biomolecule to be produced in high yields, and the absence of cytotoxicity of both peptides, the goal of this study was to rationally design AfpB::PAF26 hybrid proteins (dAfpBs) to obtain novel biomolecules and test their biotechnological production and antimicrobial properties.

## 2. Materials and Methods

### 2.1. Strains, Media and Culture Conditions

The isolate *P. digitatum* CECT 20796 (PHI26) [32] was used as a parental strain. Transformant PDSG420 is a genetically modified producer of AfpB previously reported [26]. These and all *P. digitatum* transformant strains obtained in this study were cultured on potato dextrose agar (PDA, Difco) plates for 5–7 days at 25 °C. Conidia were collected from plates, dispersed in water, filtered and counted in a hemocytometer. To analyze the growth in solid medium, 5 µL of conidial suspension (5 × 10^4^ conidia/mL) were deposited on the center of PDA plates and the diameter of growth was measured daily from 3 to 10 days. For recombinant protein production, transformant strains were inoculated at 5.5 × 10^5^ conidia/mL in *P. digitatum* minimal medium (PdMM) [25] with shaking at 25 °C for 10–12 days. For fungal transformation, vectors were propagated in *Escherichia coli* JM109 grown in Luria Bertani (LB) medium supplemented with either 25 µg/mL chloramphenicol, 50 µg/mL kanamycin or 100 µg/mL spectinomycin at 37 °C depending on the vector. *Agrobacterium tumefaciens* AGL-1 strain was grown in LB supplemented with 20 µg/mL rifampicin at 28 °C. For antimicrobial assays, *P. chrysogenum* Q176, *Botritys cinerea* CECT 2100, *Fusarium oxysporum* 4287, *Penicillium expansum* CECT 20906 (CMP-1), *Magnaporthe oryzae* PR9, *Penicillium italicum* CECT 20909 (PHI-1), and *Aspergillus niger* CBS 120.49 were incubated in PDA at 25 °C for 5–10 days depending on the fungus.

### 2.2. Rational Design of AfpB-Derived Chimeric Proteins (dAfpBs)

The hexapeptide PAF26 (RKKWFW) was inserted inside the AfpB primary amino acid sequence in different positions of the previously predicted AfpB 3D structure [27] in order to generate distinct rationally designed chimeric dAfpBs (see Results for further details). The SWISS-MODEL program [33] was used to predict the 3D structure of dAfpBs, and the models obtained were refined using the ModRefiner software tool [34] and subsequently validated with RAMPAGE [35]. The theoretical molecular mass (MM) and isoelectric point (pI) of dAfpBs were calculated with the ProtParam software of the ExPASy Proteomics Server [36]. All models were visualized with UCSF Chimera [37].

### 2.3. Construction of Plasmids for Genetic Transformation of P. digitatum

To generate all vector constructions for the recombinant production of dAfpBs in *P. digitatum*, we used the GoldenBraid-based modular cloning platform FungalBraid (FB) [26,38]. This platform was previously used to obtain the AfpB producer strain PDSG420. In silico designed dAfpB genetic sequences containing 5′/3′ DNA barcodes were provided by an external company (IDT, Integrated DNA Technologies) as synthetic genes (gBlocks). The different FB plasmids used in this work are listed in Table 1. Each genetic element was cloned into the GoldenBraid entry vector pUPD2 through restriction-ligation reactions as previously described [26,38], and constructs were verified by Sanger DNA sequencing using the primers OJM524 (5′-GCTTTCGCTAAGGATGATTTCTGG-3′) and OJM525 (5′-CAGGGTGGTGACACCTTGCC-3′). Gene fragments encoding for each of the four selected dAfpBs cloned into pUPD2 (FB040 to FB043, see Table 1) were assembled into the binary vector pDGB3α1R with FB parts of the *paf* promoter (FB029) and terminator (FB030), to generate each of the four transcriptional units (FB047 to FB050). Finally, binary assembly was conducted in the binary pDGB3Ω1 plasmid with each of these four transcriptional units with the FB plasmid encoding the hygromycin resistance cassette (*hph*) as the fungal selection marker (FB003), to obtain the plasmids for genetic transformation (FB053 to FB056) (Table 1). These binary vectors were PCR-confirmed with primers OJM533 (5′-CGAGTGGTGATTTTGTGCCG-3′) and OJM534 (5′-CCCGCCAATATATCCTGTCAG-3’).

### 2.4. A. tumefaciens Mediated Transformation of P. digitatum

The four binary plasmids (FB053/FB054/FB055/FB056) were introduced into *A. tumefaciens* AGL-1 strain by electroporation. *P. digitatum* CECT 20796 was genetically transformed through *A. tumefaciens*-mediated transformation (ATMT) as described [39] with some modifications [40]. Monosporic hygromycin resistant strains were isolated and maintained. Transformants were confirmed with distinct combinations of PCR reactions following routine procedures [19,22,25,26].

### 2.5. Production of dAfpBs in P. digitatum Transformants and Purification of the Recombinant Proteins

The recombinant production of each dAfpB was tested in different transformant strains grown in PdMM for up to 10 days at 180 rpm (final concentration 5.5 × 10^5^ conidia/mL). Cell-free supernatants were collected and tenfold concentrated to monitor protein accumulation by SDS-PAGE electrophoresis [41] using SDS-16% polyacrylamide gels calibrated with prestained SeeBlue^®^ protein standard (Invitrogen) and Coomassie stained. Samples were compared with purified AfpB from the PDSG420 strain. The best dAfpB producers were chosen for the bulk production and purification of each dAfpB. The protein dAfpB9 was purified from cell-free supernatants of *P. digitatum* transformant strains grown in PdMM for 10 days, similarly to previously reported for AfpB [19]. Briefly, clarified supernatants were dialyzed (2 kDa molecular weight cut-off; Sigma-Aldrich) against 10 mM phosphate buffer pH 6.6. Dialyzed solutions were applied to an AKTA Purifier system equipped with a 6 mL RESOURCE S cation exchange chromatography column (GE Healthcare). Protein was eluted with a linear NaCl gradient from 0 to 1 M in the same buffer. Protein containing fractions were pooled and dialyzed against Milli-Q water. Protein concentration was determined spectrophotometrically (A_280_) considering the molar extinction coefficient (dAfpB9_Ɛ280_ = 1.66). Homogeneity of the purified protein was checked with SDS-PAGE electrophoresis and Coomassie blue staining.

### 2.6. Matrix-Assisted Laser Desorption/Ionization–Time-of-Flight Mass Spectrometry (MALDI-TOF MS)

Analyses were performed in the proteomics facility at the SCSIE of the University of Valencia. Proteins were identified and confirmed by peptide mass fingerprinting (PMF). Samples from bands excised from either SDS-PAGE gels or purified proteins were digested with trypsin and the resulting mixtures were analyzed on a 5800 MALDI TOF/TOF (AB Sciex), with a mass accuracy of 50 ppm in positive reflectron mode (3000 shots every position). Five of the most intense precursors (according to the threshold criteria: minimum signal-to-noise: 10, minimum cluster area: 500, maximum precursor gap: 200 ppm, maximum fraction gap: 4) were selected for every position for the MS/MS analysis. MS/MS data was acquired using the default 1 kV MS/MS method. The MS and MS/MS information was sent to MASCOT via the Protein Pilot (AB Sciex).

### 2.7. Fungal Growth Inhibition Assays

Growth inhibition assays of AfpB and dAfpB9 were conducted in 96-well flat bottom microtiter plates (Nunc) in a total volume of 100 µL. Fifty µL of 2× concentrated conidia (5 × 10^4^ conidia/mL) in 10% potato dextrose broth (PDB) containing 0.02% (*w*/*v*) chloramphenicol to avoid bacteria contamination were mixed in each well with 50 µL of 2× concentrated protein from serial twofold dilution (from 1 to 128 µg/mL, final concentration). Samples were prepared in triplicates. Plates were statically incubated for 72–96 h at 25 °C. Growth was determined every 12–24 h by measuring the optical density at 600 nm (OD_600_) using a Fluostar Omega plate spectrophotometer (BMG Labtech), and the OD_600_ mean and standard deviation (SD) were calculated. Dose-response curves were generated from measurements after 72 h. The experiments were repeated at least twice. Minimum inhibitory concentration (MIC) is defined as the protein concentration that completely inhibited growth in all the experiments performed. Low magnification images of each well in the 96-well plate were visualized under the microscope (E90i, Nikon Chiyoda, TO, Japan), captured by the NIS-Elements BR v2.3 software (Nikon) and processed by the FIJI software [42]. Experiments were repeated at least twice for each fungus.

### 2.8. Fungicidal Activity Assays

Fungicidal activity of the synthetic peptide PAF26 and proteins AfpB and dAfpB9 were assessed by incubation of *P. digitatum* conidia (2.5 × 10^4^ conidia/mL) with two protein/peptide concentrations (MIC and 4× MIC) in sterile Milli-Q water for 24 h at room temperature and 60 rpm. Treatments were prepared in triplicate. After each treatment, samples were serially diluted and spread onto PDA plates that were incubated for 72 h at 25 °C to count colony-forming units (CFU). Data were used to calculate the number of viable conidia after each peptide treatment compared to the untreated control. Experiments were repeated at least twice.

## 3. Results

### 3.1. In Silico Design of dAfpBs and Structural Modeling

**Figure 1:**
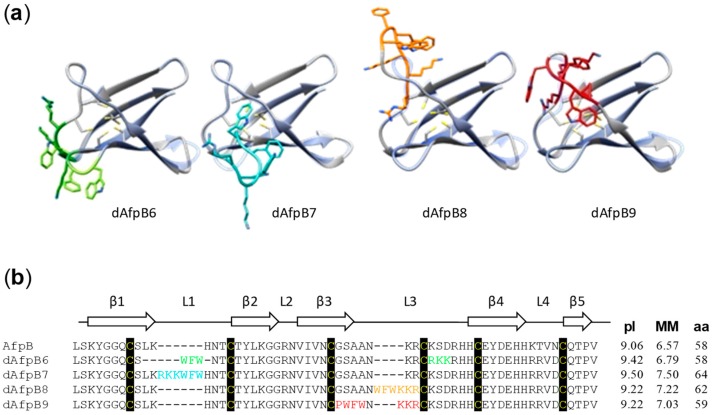
Three-dimensional structures of the four different dAfpBs and their sequence alignment. (**a**) Graphical representation of dAfpB6, dAfpB7, dAfpB8, and dAfpB9 (grey) overlapped with native AfpB (blue). PAF26 insertions/substitutions are highlighted in a different color for each model (green, blue, orange, and red for dAfpB6, dAfpB7, dAfpB8, and dAfpB9, respectively); (**b**) sequence alignment, isoelectric point (pI), molecular mass (MM, in kDa), and number of amino acids (aa) of the four different dAfpBs. Insertions and/or substitutions are highlighted following the color code in (**a**). The conserved cysteine pattern is shadowed in black.

To obtain chimeric AfpB::PAF26 proteins, we rationally designed and evaluated a number of different models. To design these chimeras, we used the modeled structure of AfpB [27] as well as the amino acid sequence alignment of class B AFP proteins known to date (Supplementary Figure S1). The models evaluated different alternatives such as: (i) insertion or substitution of the PAF26 sequence in the AfpB sequence; (ii) direct or reverse orientation; or (iii) continuous PAF26 sequence or split in two. PAF26 was located in two different regions of AfpB that concentrate most of the protein variability within class B, suggesting that these are more permissive to sequence variation (Supplementary Figure S1). The first region was the γ-core motif present around loop 1 (L1) of the native AfpB. The second corresponds to loop 3 (L3), the most cationic, exposed, and flexible loop of AfpB and in which antifungal activity was previously demonstrated in a synthetic peptide derived from this region [27]. Four different models named dAfpB6, dAfpB7, dAfpB8 and dAfpB9 finally passed the validation pipeline. The 3D structure of these models and their sequence alignments are shown in Figure 1. All models were refined and subsequently evaluated with the RAMPAGE software (see Materials and Methods). This software predicts for each model the amino acids that are located in energetically favored, allowed, and not allowed regions attending to the RAMACHANDRAN Plot (Supplementary Figure S2). The predicted structures of the four models obtained do not vary significantly with respect to the native AfpB structure in regions other than those modified. Results of the evaluation of these models are shown in Table 2. All models showed a high percentage of amino acids in the energetically favored zone (>96%) and none are situated in a not allowed region.

The protein dAfpB6 is an example of the “split and substitution strategy”, the LKH residues in positions 10–12 (L1) in AfpB were substituted for the aromatic domain of PAF26 (WFW), and the KSD residues in positions 37–39 (L3) for the cationic domain (RKK). Although, in this model, the two domains of PAF26 are separated in the AfpB primary sequence, they come close in space in the 3D structure (Figure 1a). The resulting model has a MM of 6.79 kDa, very similar to the native AfpB, and a theoretical pI of 9.42 (Figure 1b). In order to provide antifungal properties to the inactive γ-core motif of AfpB [27], PAF26 was directly inserted in position 11 to obtain dAfpB7, an example of the “forward insertion strategy”. This model has the highest theoretical pI (9.50) and MM (7.50 kDa), since the whole sequence of PAF26 is inserted and no substitutions are taking place. The dAfpB8 resulted from a “reverse insertion strategy”, a partial reverse sequence of PAF26 (WFWK) was inserted in position 33 (L3) of AfpB to reconstitute the PAF26 sequence with the KR at positions 34–35 of AfpB. The pI of dAfpB8 is 9.22 and the MM is 7.22 kDa. Finally, dAfpB9 was the more complex model; positions 30–32 of AfpB were substituted by the amino acids WFW of PAF26, and K was inserted at position 34. As occurs with dAfpB8, the KR at positions 34–35 of AfpB reconstituted the cationic domain of PAF26 in reverse orientation. It has to be noted that the highly conserved amino acid asparagine (N) (Supplementary Figure S1) was maintained at position 33 and separates the PAF26 sequence in the primary structure but allows close proximity of the PAF26 residues in the 3D model. In addition, the glycine (G) residue at position 29 of AfpB was changed for proline (P), which is a residue present in most of the class B proteins at that position (Supplementary Figure S1). This latter substitution resulted in a more energetically favored model, leading to a final model with a theoretical pI of 9.22 and a MM of 7.03 kDa.

### 3.2. Recombinant Production, Identification, and Purification of dAfpBs in P. digitatum

**Figure 2:**
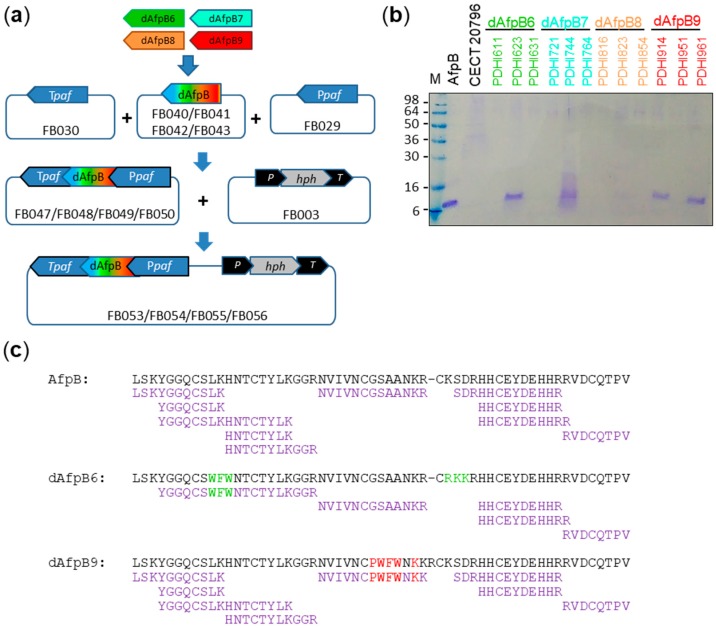
Production and identification of dAfpBs in *P. digitatum.* (**a**) Schematic diagram of the modular assembly of each different dAfpB genetic sequence into pUPD2 vectors with FB030 and FB029 (*paf* promoter and terminator sequences, respectively) to obtain pDGBα plasmids FB047, FB048, FB049, and FB050, and a subsequent binary assembly of these plasmids with FB003 to obtain final pDGBΩ vectors FB053, FB054, FB055, and FB056; (**b**) representative SDS-PAGE analysis of the production of dAfpBs in 10× culture supernatants of *P. digitatum* after 9 days of incubation. Two µg of pure AfpB were added as control. M: Seeblue^®^ pre-stained protein standard. (**c**) Peptide fingerprint of dAfpB6 and dAfpB9 obtained from trypsin-digested gel bands. The peptide fingerprint of native AfpB was added as control. Peptides obtained covered 96%, 86% and 94% of AfpB, dAfpB6 and dAfpB9 primary sequences, respectively.

The recombinant production of the four different dAfpBs was achieved with the adaptation of the *P. chrysogenum*-based *paf* expression system [25] to the FungalBraid modular cloning platform [26] (Figure 2a). The universal modular FB parts containing the promoter (FB029) and terminator (FB030) of the *paf* gene were assembled to FB parts coding for each of the four dAfpBs (FB040 to FB043), and the resulting transcriptional units (FB047 to FB050) to the universal FB003 *hph* resistant marker. The resulting binary vectors (FB053 to FB056) were directly used for ATMT of *P. digitatum*.

Genetically transformed hygromycin-resistant *P. digitatum* strains (between four and six depending on the construct) were tested for dAfpBs production. A representative SDS-PAGE gel of 10× supernatants of *P. digitatum* transformants is shown in Figure 2b. Three out of the four different dAfpBs could be produced in detectable amounts under our assay conditions. Only one producer clone was identified for dAfpB6 (PDHI623) and dAfpB7 (PDHI744) (Figure 2b. However, the protein band produced by PDHI744 (dAfpB7) showed diffuse staining and could never be detected or purified beyond these pilot experiments, and therefore, we assumed it was degraded during production. None of the six strains transformed with the dAfpB8 gene construct was able to produce any distinctive protein bands of the expected size (7 kDa) in detectable amounts. Finally, three dAfpB9-transformed clones successfully produced dAfpB9 (Figure 2b. To verify the identity of the recombinant dAfpB6 and dAfpB9, PMF analyses of the corresponding trypsin-digested gel bands were carried out, and the identity of both proteins were confirmed (Figure 2c). For dAfpB6, 86% of the sequence was covered by PMF, whereas sequence coverage for dAfpB9 was 94%. Note that the central uncovered regions for both proteins are rich in R and K, which are the target amino acids for trypsin, making it difficult to detect the small resulting digested peptides with PFM. In fact, a control PMF run on AfpB revealed that the CK residues at positions 36–37 are not covered either in the native protein (Figure 2c).

**Figure 3:**
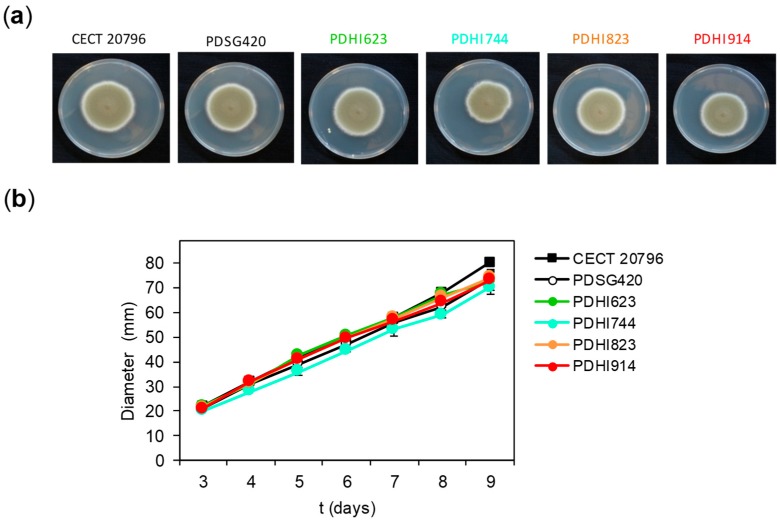
Phenotypical characterization of the *P. digitatum* dAfpBs transformant strains. Colony morphology (**a**) and colony diameter measurement (**b**) on PDA plates of the *P. digitatum* dAfpBs transformant strains PDHI623 (dAfpB6), PDHI744 (dAfpB7), PDHI823 (dAfpB8), and PDHI914 (dAfpB9). CECT 20796 and PDSG420 strains were added as controls. Plotted data are mean values ± SD of triplicate samples.

**Figure 4:**
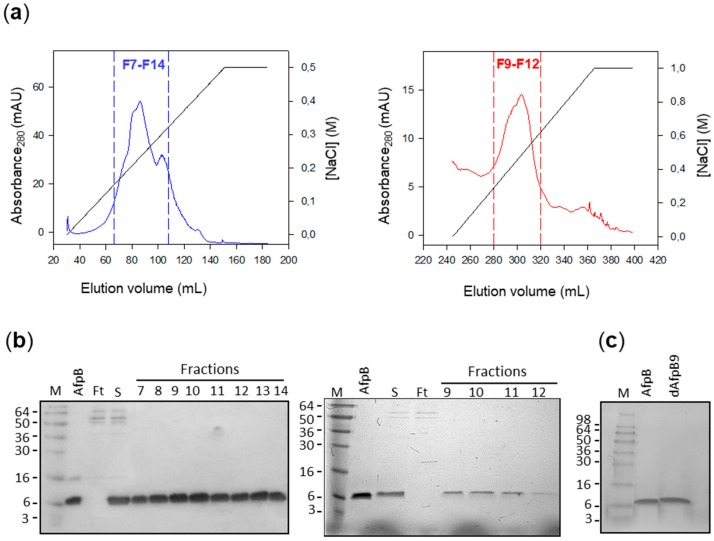
Purification of native AfpB and dAfpB9 by cation exchange chromatography. (**a**) Chromatograms of native AfpB (dark blue) and dAfpB9 (red) purification. NaCl gradient is represented with a black line. The analyzed fractions are represented between discontinuous lines; (**b**) SDS-PAGE analyses of chromatographic fractions obtained from AfpB purification (left) and dAfpB9 (right). Two µg of pure AfpB were added as control. Ft: Flow-through. S: Initial sample. M: Seeblue^®^ pre-stained protein standard. (**c**) SDS-PAGE of 2 µg of pure AfpB and dAfpB9.

The growth of the transformant strains was indistinguishable from that of the wild type strain CECT 20796 and the previous AfpB producer PDSG420 (Figure 3). The clones PDHI623 and PDHI914 were chosen for dAfpB6 and dAfpB9 production and purification, respectively. Native AfpB was purified as an internal control of the chromatography conditions and for yield comparison. As expected for their physico-chemical properties, both AfpB and dAfpB9 were successfully purified by one-step cation exchange chromatography using the standard 10 mM phosphate buffer pH 6.6 (Figure 4). Protein dAfpB9 was purified with a yield of 3.6 mg/L, whereas the yield of native AfpB was 24 mg/L, similar to that of the previously reported yields for AfpB in other *P. digitatum* AfpB producer strains [19].

However, the theoretical physico-chemical properties of dAfpB6 (pI = 9.42) did not correlate with the empirical determinations, since dAfpB6 was not retained in the cation exchange chromatography column under the same conditions as AfpB and dAfpB9. Other chromatographic conditions were tested in order to achieve the successful purification of dAfpB6, but all of them were unsatisfactory and the purification of dAfpB6 had to be abandoned.

### 3.3. Antifungal Activity of dAfpB9 Against Filamentous Fungi

**Figure 5:**
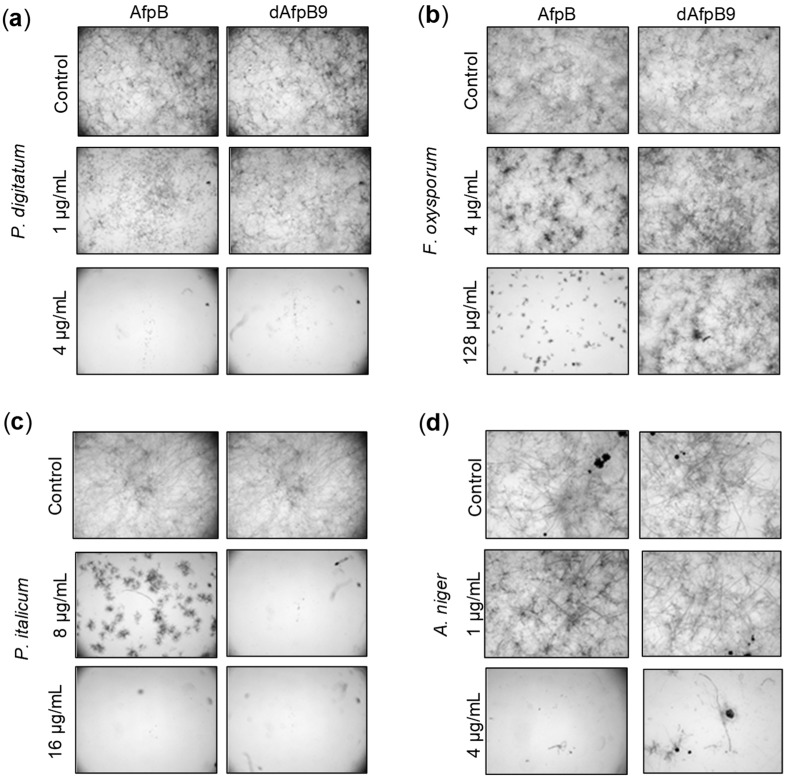
Antifungal activity of dAfpB9 against filamentous fungi. Microscopy images of representative wells for the comparison of the antifungal activity of native AfpB and dAfpB9 against *P. digitatum* (**a**), *F. oxysporum* (**b**), *P. italicum* (**c**), and *A. niger* (**d**) after 7 days of growth at the concentrations shown.

The antifungal activity of dAfpB9 was tested and compared to that of the native AfpB protein against a selection of filamentous fungi. This selection includes: *P. digitatum*, the fungus in which AfpB was identified, and against which it is highly active [19]; several plant pathogens like the citrus and pome postharvest pathogens *P. italicum* and *P. expansum*, the polyphagous *B. cinerea*, the rice blast fungus *M. oryzae*, the soilborne plant pathogen *F. oxysporum*; and industrially relevant GRAS organisms such as *P. chrysogenum*, and *A. niger.* Differences in antifungal activity were observed between AfpB and dAfpB9 (Table 3 and Figure 5). In *P. digitatum*, *B. cinerea*, *P. expansum*, *M. oryzae* and *A. niger*, dAfpB9 showed antifungal potency similar to that of the native AfpB, and MIC values are maintained (Table 3). Protein dAfpB9 showed a slightly better antifungal activity against *P. italicum* than that of the native AfpB, with a MIC value of 8 µg/mL compared to 16 µg/mL of AfpB (Table 3). Microscopic observations confirmed this result (Figure 5); the growth of *P. italicum* was severely affected at 8 µg/mL of AfpB but completely inhibited at the same concentration of dAfpB9. On the contrary, in the case of *P. chrysogenum*, the new dAfpB9 was less active than the native AfpB, with a twofold reduction of the MIC value to 8 µg/mL, the same value observed for *P. italicum* (Table 3). Finally, the very limited activity of AfpB towards *F. oxysporum* disappears in the case of dAfpB9 (Figure 5).

### 3.4. Fungicidal Activity

**Figure 6:**
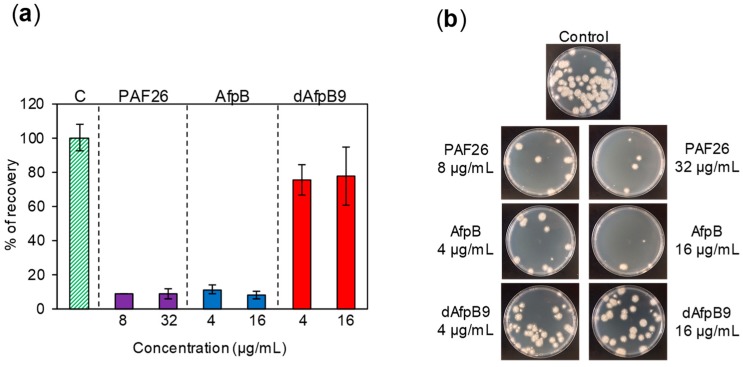
Study of AfpB and dAfpB9 fungicidal activity. (**a**) Percentage of recovery of viable conidia after 24 h of protein treatment at the concentrations shown (MIC and 4× MIC) and after 72 h of conidia incubation in PDA. PAF26 was added as a positive control for fungicidal activity; (**b**) representative images of conidia viability after 24 h of protein treatment and 72 h of conidia incubation on PDA plates at 25 °C.

The inhibitory activity of the hexapeptide PAF26 was accompanied by fungicidal activity against the quiescent conidia of *P. digitatum*, which were killed and did not recover after treatment with this peptide [10]. The fungicidal activity of AfpB has not been evaluated to date. In this work, the fungicidal activity of AfpB and dAfpB9 were studied and compared, and PAF26 was added as control. Fungicidal activity assays revealed that AfpB shows killing capability against conidia of *P. digitatum* similar to that of PAF26 when co-incubated at 4 and 16 µg/mL for 24 h (Figure 6). However, dAfpB9 shows no fungicidal activity despite the insertion of PAF26, since the number of viable conidia after protein treatment is similar to that of the untreated control (Figure 6). This result suggests that determinants of the fungicidal activity of AfpB locate in loop L3, where the modifications of dAfpB9 occur. In addition, it demonstrates that the properties of two different AMPs (PAF26 and AfpB) are not necessarily maintained in a chimeric peptide designed from them.

## 4. Discussion

In this work, we explored the possible improvement of the *P. digitatum* AfpB antifungal and/or fungicidal activity by inserting the antifungal peptide PAF26 into the AfpB primary sequence. We rationally designed four different dAfpBs and chose *P. digitatum* as biofactory for recombinant protein production, since this fungus had been successfully used for the biotechnological production of other AFPs, such as PAF from *P. chrysogenum* and its own AfpB in high yields (80 and 20 mg/L, respectively) [19,25]. In previous studies, we used synthetic peptides to identify two antifungal motifs within the AfpB primary sequence, which are located at loops L2 and L3 [27]. Loop L3 seems to be relevant for the antifungal activity of AFPs, since a synthetic peptide derived from the L3 of PAF also showed antifungal activity [27], and protein mutations in this loop negatively affected its antifungal properties [18,25]. On the other hand, the γ-core motif from AfpB located at loop L1 did not show antifungal activity [27], analogous to the recently described γ-core from the *Neosartoria fischeri* NFAP2 [43], and thus was suggested to have a structural function in AfpB [27]. On the contrary, the γ-core motif of PAF has antifungal activity by itself [44]. In plant defensins, which are other cysteine-rich antifungal proteins structurally related to fungal AFPs, some γ-core motifs have been reported to have antifungal activity, as it is the case of MtDef4 from *Medicago truncatula* [45] and BhDef2 from *Brassica hybrid* [46], while in other cases a structural role for this γ-core is proposed [45].

The antifungal properties of PAF26 against filamentous fungi were previously well established, with MIC values ranging from 8 µM (8 µg/mL) in the case of *P. digitatum*, *A. niger*, or *B. cinerea*, to 32 µM (32 µg/mL) for *P. expansum* [19,47]. In the present work, we aimed to provide the AfpB γ-core motif with antifungal activity by incorporating PAF26 into the loop L1, obtaining the models dAfpB6 and dAfpB7; and on the other hand, to increase the antifungal potency of AfpB by adding PAF26 to loop L3 in models dAfpB8 and dAfpB9. In a recent study, a 14 residue cationic peptide that spans the PAF γ-core showed antifungal and fungicidal activity against the yeast *Candida* comparable to the native PAF [44]. This is in marked contrast with an 11 residue peptide derived from the γ-core of AfpB that is less cationic and did not show antifungal activity against filamentous fungi [27]. Moreover, peptide sequence variants were designed that increased the cationicity and improved the activity of peptides derived from the γ-core of the PAF protein, and when these sequence modifications were incorporated to the native PAF they also resulted in a clear improvement of the PAF variants against *Candida albicans*, with a MIC reduction from 5 to 1.3 µM [44]. Unfortunately, our designs that aimed at the modification of the AfpB γ-core could not be tested due to failure in their production and/or purification. However, the unique successful insertion of PAF26 in dAfpB9, although it improved the cationicity of the resulting protein, did not substantially improve AfpB antifungal potency. It is obvious that further comparative studies with the same microorganisms and additional protein designs are needed to shed light on these discrepancies between the AfpB and PAF studies. However, it must also be taken into account that AfpB is clearly more active (>10 times) than PAF against most of the filamentous fungi tested in parallel experiments, for instance with a MIC value in µM units of 0.6 (3–4 µg/mL) versus 8.0 (50 µg/mL) against *P. digitatum* [19]. This is a remarkably high activity for AfpB, which leads to the hypothesis that a threshold or limit of high activity has been reached in AfpB and, therefore, substantial improvements as the ones attempted in our study, might not be successful.

A negative result of our study that, however, leads to very significant conclusions is that the insertion of the fungicidal PAF26 sequence into the fungicidal AfpB protein—as in dAfpB9—caused an unexpected and almost complete loss of fungicidal activity towards *P. digitatum* conidia while the fungistatic activity remained the same. This fact suggests that the insertion of an antifungal peptide, in this case of PAF26, does not always lead to the improvement of the antifungal properties in the resulting hybrid protein, and indirectly, this result led us to map determinants of AfpB fungicidal motif in loop L3.

The place where PAF26 is inserted in AfpB seems to be determinant for protein stability and accumulation. From the four dAfpBs designed in this work, only dAfpB6 and dAfpB9 could be successfully produced in *P. digitatum* culture supernatants, while dAfpB7 showed signals of degradation and dAfpB8 was undetectable in the supernatants of all the strains tested (Figure 2). Probably, dAfpB7 and dAfpB8 instability might be due to the increase in the size of the modified γ-core motif in dAfpB7 and to the increase of loop L3 in dAfpB8 when PAF26 is directly inserted in both regions. These insertions generate larger and more prominent surface-exposed loops in the resulting proteins that, although our in silico modeling predicted not detrimental, could be more accessible to proteases in vivo. Something similar was observed with a protein variant of the *N. fischeri* antifungal protein NFAP. This variant was completely degraded when all the cysteines of the native protein were replaced by tyrosine residues, which resulted in the apparition of a big surface-exposed loop that was more accessible to proteases [48]. On the other hand, dAfpB6, in which PAF26 was divided into its two differentiated domains (the aromatic WFW and the cationic RKK) and were inserted in loops L1 and L3 of AfpB, respectively, could be produced but not successfully purified. In this case, the theoretical physico-chemical properties of dAfpB6 did not correlate with empirical determinations, since this protein was not retained in the chromatography column under the same conditions as AfpB and dAfpB9, with very similar isoelectric points, size and predicted structure. The apparent molecular weight of dAfpB6 in SDS-PAGE gels and the peptide fingerprint results indicate that dAfpB6 is correctly translated (Figure 2b,c). However, the lack of the three first amino acids at the N-terminus that were not detected by PMF (Figure 2c) could also indicate a propensity to degradation.

The dAfpB9 could be successfully purified, although the yield obtained (3.6 mg/L) is six times lower than that of the native AfpB. A lower protein stability or a possible degradation could be the main reason for this low yield obtained for dAfpB9, since the antifungal activity of this protein does not differ much from that of the native AfpB. However, it must positively be taken into account that this yield is yet better than those obtained with other AFPs naturally produced by fungi, as it is the case, for example, of NFAP and NFAP2 from *N. fischeri*, with yields of 1.25 and 0.368 mg/L, respectively [23,49].

Our overall results demonstrate that dAfpB9 does not improve the MIC values of AfpB against the producer *P. digitatum*, or other filamentous fungi like *B. cinerea*, *P. expansum*, or *A. niger*. A slight improvement of the antifungal activity of dAfpB9 was only observed for *P. italicum* (twofold reduction of MIC). This occurs despite the fact that PAF26 is less active against *P. italicum* than against *P. digitatum* [47]. On the other hand, dAfpB9 is less active than AfpB towards *P. chrysogenum* and *M. oryzae.* Further studies are needed to determine whether these minor changes in activity reflect subtle differences in the mode of action of the AfpB and dAfpB9 towards the different fungi.

In summary, we can conclude that the rational design of AfpB variants based on the unrelated PAF26 hexapeptide did not substantially improve the AfpB antifungal potency. However, we reported the fungicidal activity of AfpB for the first time, and were able to map a fungicidal motif located at loop L3 of AfpB. Our study provides information on the potential improvement of AFPs by rational design, and further studies/combinations might provide new chimeric proteins with improved properties.

## Figures and Tables

**Table 1:** Plasmids Used in this Study.

Vector ID	Genetic Element/Assembly	Plasmid	Reference
FB003	P*trpC* + *hph* + T*tub*	pDGB3α2	[26]
FB029	P*paf*	pUPD2	[26]
FB030	T*paf*	pUPD2	[26]
FB040	*dafpB6*	pUPD2	This work
FB041	*dafpB7*	pUPD2	This work
FB042	*dafpB8*	pUPD2	This work
FB043	*dafpB9*	pUPD2	This work
FB047	FB029 + FB040 + FB030	pDGB3α1R	This work
FB048	FB029 + FB041 + FB030	pDGB3α1R	This work
FB049	FB029 + FB042 + FB030	pDGB3α1R	This work
FB050	FB029 + FB043 + FB030	pDGB3α1R	This work
FB053	FB047 + FB003	pDGB3Ω1	This work
FB054	FB048 + FB003	pDGB3Ω1	This work
FB055	FB049 + FB003	pDGB3Ω1	This work
FB056	FB050 + FB003	pDGB3Ω1	This work

**Table 2:** Results of the evaluation (number of amino acids) of each different dAfpB model obtained by rational design.

Model ID	Favored	Allowed	Not Allowed	Total	Source
AfpB	57	1	0	58	[27]
dAfpB6	57	1	0	58	This work
dAfpB7	63	1	0	64	This work
dAfpB8	60	2	0	62	This work
dAfpB9	58	1	0	59	This work

**Table 3:** Minimum inhibitory concentration (MIC) (µg/mL) of AfpB and dAfpB9.

Microorganism	AfpB	dAfpB9
*P. digitatum*	4	4
*P. chrysogenum*	4	8
*B. cinerea*	16	16
*P. expansum*	4	4
*P. italicum*	16	8
*F. oxysporum*	>128	NI ^1^
*M. oryzae*	>128	>128
*A. niger*	4	4

^1^ NI: Not inhibitory at the maximum concentration tested (128 µg/mL).

## Supplementary Materials

The following are available online at http://www.mdpi.com/2076-2607/6/4/106/s1, Figure S1: Sequence alignment of class B AFPs and AFP-like proteins of filamentous fungi, Figure S2: Ramachandran Plot of the structural models of dAfpBs.
