# Supplementary material for: Rational Design and Biotechnological Production of Novel AfpB-PAF26 Chimeric Antifungal Proteins
Source: Microorganisms. 2018 Oct 15;6(4):106. doi: 10.3390/microorganisms6040106 (PMC6313716; doi:10.3390/microorganisms6040106)
Supplement: Supplementary file 1 [file microorganisms-06-00106-s001.pdf]

Supplementary Figures

Microorganisms

Rational Design and Biotechnological Production of New AfpB-PAF26 Chimeric Antifungal Proteins

Marcos Heredero †, Sandra Garrigues †, Mónica Gandía, Jose F. Marcos *, and Paloma Manzanares.

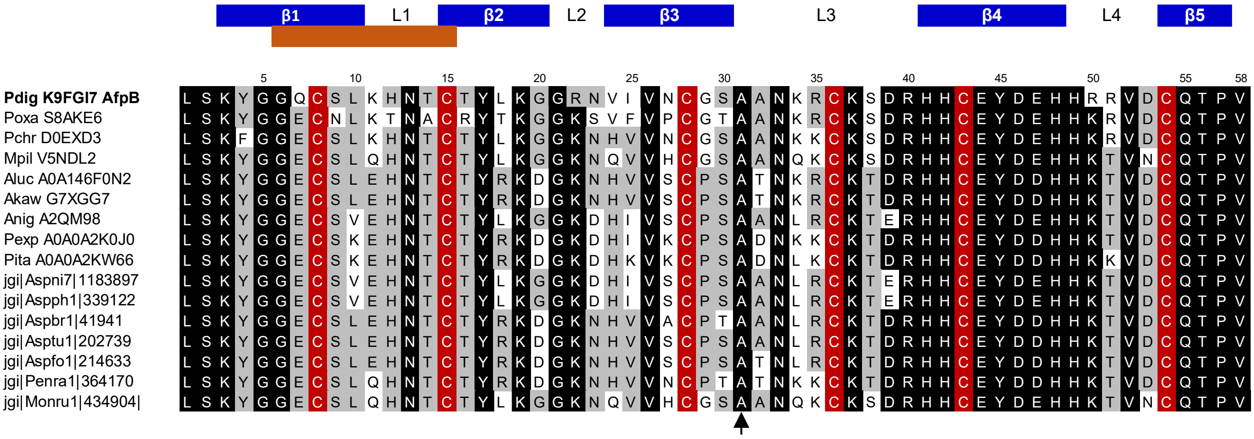

Supplementary Figure S1. Sequence alignment of class B AFPs and AFP-like proteins of filamentous fungi. The amino acid sequence of native AfpB from *P. digitatum* is at the top and is highlighted in black. The cysteine conserved pattern is shadowed in red. Strongly conserved amino acids are highlighted in black. Other conserved amino acids are shadowed in grey. Blue bars at the top represent the predicted secondary structure of AfpB (β: beta strand; L: loop). Brown bar at the top shows the position where the conserved γ-core motif is located in class B AFPs. The black arrow shows the amino acid position where Glycine and Proline residues are conserved.

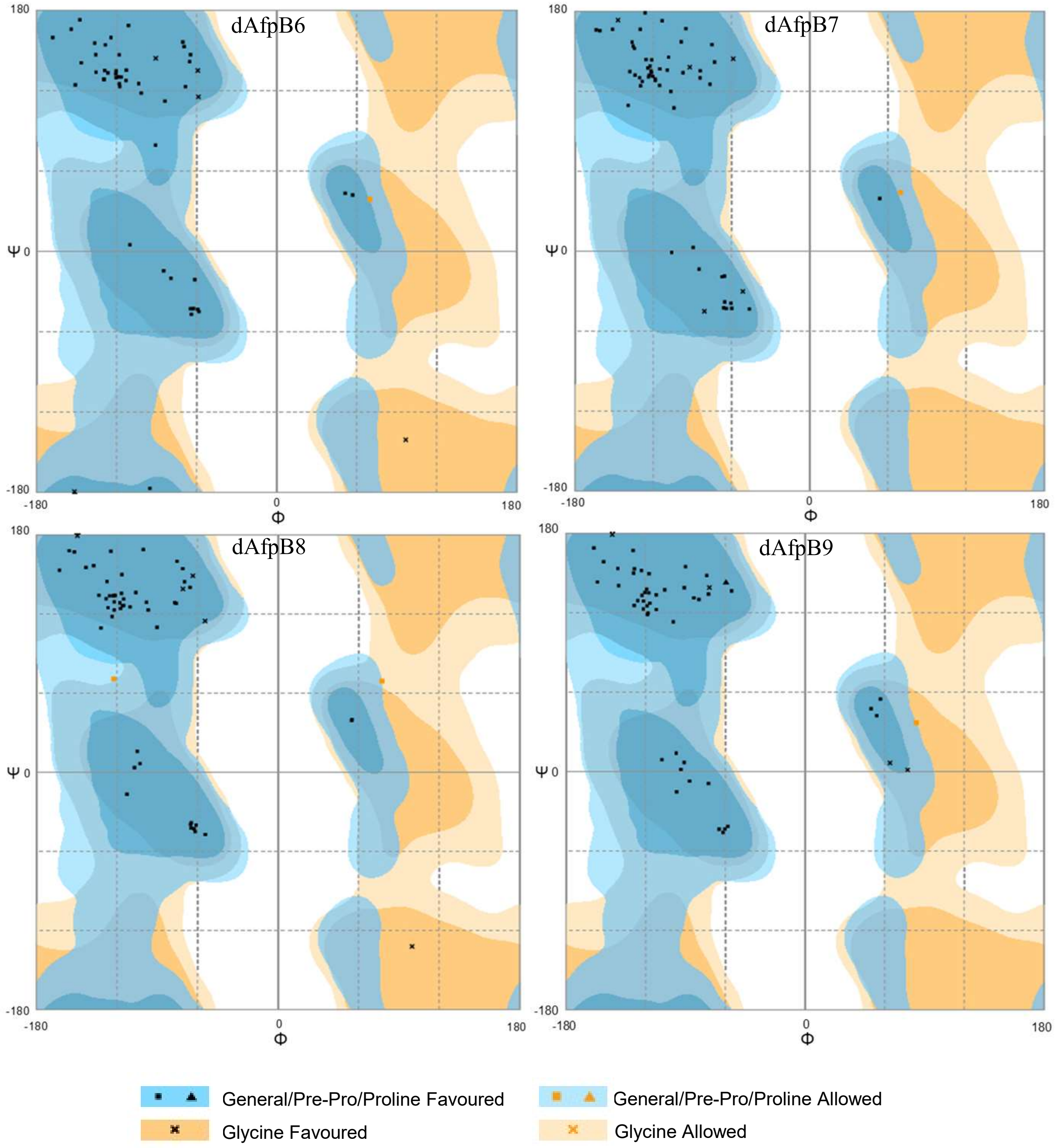

Supplementary Figure S2. Ramachandran Plot of the structural models of dAfpBs. Visualization of the energetically allowed regions for backbone dihedral angles ψ against φ of the amino acid residues in dAfpB6, dAfpB7, dAfpB8, and dAfpB9 using RAMPAGE software tool. General amino acids are represented with a square. Proline residues are represented with a triangle. Glycine residues are represented with a cross. Energetically favored and allowed regions for all amino acids (excluding Glycine) are represented in dark and light blue respectively. Energetically favored and allowed regions for Glycine are represented in dark and light orange, respectively. Amino acids located in energetically favored regions are colored in black. Amino acids located in energetically allowed regions are highlighted in orange. In dAfpB6, 57 amino acids out of 58 (98%) are located in energetically favored regions, while 1 amino acid (Arg40) is located in an energetically allowed region. In dAfpB7, 63 amino acids out of 64 (98%) are located in energetically favored regions, while 1 amino acid (Arg46) is located in an energetically allowed region. In dAfpB8, 60 amino acids out of 62 (96%) are located in energetically favored regions, while 2 amino acids (Ala31 and Arg44) are in energetically allowed regions. In dAfpB9, 58 amino acids out of 59 (98%) are located in energetically favored regions, while 1 amino acid (Arg41) is in an energetically allowed region.
